# The fish that rashes: Contact urticaria syndrome induced by recreational fishing

**DOI:** 10.1016/j.jdcr.2025.10.004

**Published:** 2025-10-10

**Authors:** Sarah Woodside, Liam Deegan, Lourdes Lopez, Jared Roberts

**Affiliations:** a Department of Dermatology, San Antonio Uniformed Services Health Education Consortium, San Antonio, Texas; b Department of Health Education and Training, Tripler Army Medical Center, Honolulu, Hawaii

**Keywords:** contact urticaria syndrome, contact urticaria, dermatitis, fishing, occupational, protein contact dermatitis, urticaria

## Introduction

Contact urticaria can occur in an immunologic or nonimmunologic fashion in response to direct contact with environmental allergens.^1^ The immunologic response is an immunoglobulin E (IgE) mediated type I hypersensitivity reaction that requires prior sensitization to an allergen.^2^^,^^3^ Upon re-exposure, the preformed antibodies will then trigger the release of mediators such as histamine from mast cells. Classically, wheals and erythema will develop within 10 to 30 minutes of exposure to the allergen, last for minutes to hours, and resolve in <24 hours.^1^ The most common sites for contact urticaria are the hands, wrists, arms, and face.^4^ Some risk factors for poor prognosis with contact urticaria are male sex, severe symptoms, and personal history of atopy. The nonimmunologic type of contact urticaria is commonly related to exposures to toxins or irritants that directly trigger the skin reaction without prior exposure.^1^

Protein contact dermatitis (PCD) is another dermatologic entity theorized to result from type I and IV hypersensitivity reactions to proteins.^2^^,^^5^ Unlike contact urticaria, PCD presents with more eczematous changes. Initially, there is an acute phase of wheals, erythema, or angioedema followed by chronic hand dermatitis with erythema, lichenification, fissures, and scaling.^1^ These findings are more often observed on fingertips, occasionally extending to the wrists. The combined findings of contact urticaria and PCD have been described as “contact urticaria syndrome,” which is a term first coined in 1975.^1^ Although contact urticaria in the general population is estimated at 1% to 3%,^1^ the number of cases of contact urticaria syndrome is unknown. There are no reported cases of this syndrome related to recreational fishing upon literature review.

## Case report

A 32-year-old military pilot and recreational fisherman presented with a 2-year history of episodic finger swelling, burning, tingling, erythema, and subsequent desquamation. These symptoms consistently emerged during recreational fishing activities and were particularly triggered by direct handling of freshly caught fish. Although the acute rash would improve within 3 hours, residual fingertip erythema and skin peeling persisted for 3 to 4 days. These symptoms occurred anytime he handled fresh-caught fish near his home in Corpus Christi, Texas, regardless of whether he was in saltwater or freshwater conditions. Attempts at using different fishing rods, reels, and tackle did not provide relief. He also tried gloves of various materials including nitrile gloves and mesh woven gloves that helped when handling and filleting the fish, although he noted symptoms when fish bones punctured through the glove materials. Importantly, the patient reported no adverse effects when consuming the fish.

**Fig 1:**
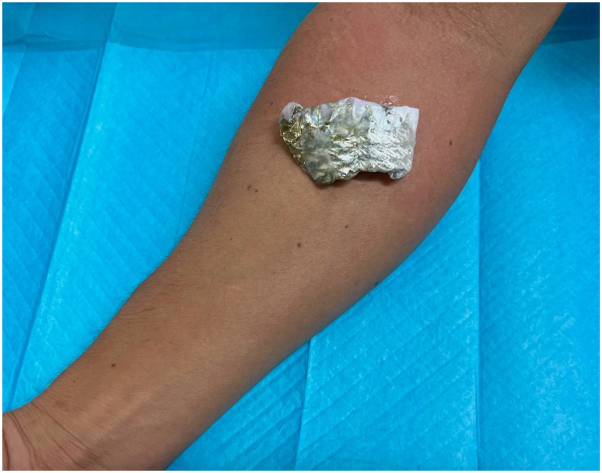
Freshly caught fish skin applied to antecubital fossa with near-immediate production of erythema, wheels, and pruritus.

**Fig 2:**
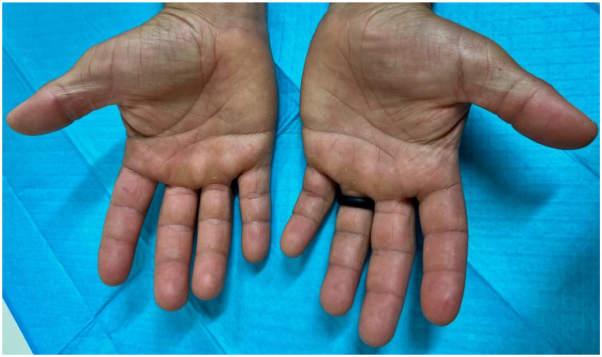
Prick testing of fingertip elicited erythema, swelling, and subsequent discomfort.

**Fig 3:**
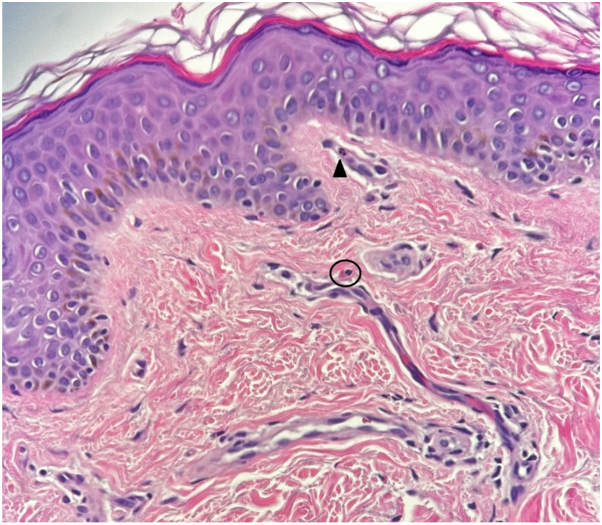
Histopathology of the biopsy specimen shows papillary dermal edema and diapedesis of neutrophils (*arrowhead*) and eosinophils (*circled*), which is consistent with a diagnosis of urticaria.

The patient’s symptoms were evaluated using a prick test panel administered by the allergy and immunology service. Reactivity to 58 allergens including trees, weeds, molds, grasses, and animals was tested but unfortunately fish proteins were not included in the standard panel. These prick test results showed an end point reactivity of 5 or 6 out of 6 on all allergens tested. An autoimmune work up was performed including tryptase and IgE levels with results notable for a mild elevation in IgE levels at 224.9 IU/mL (reference range of 0.0-100.0 IU/mL). An open application test was completed in the dermatology clinic with near-immediate wheel formation and erythema upon application of freshly caught fish skin on the antecubital fossa of his right arm (Fig 1). Additionally, the patient pricked his finger with a fish bone directly after being removed from the fish, resulting in swelling and redness of his distal digits (Fig 2). The urticarial reaction on the antecubital fossa improved within 30 minutes after the removal of the fish skin and cleansing of the area with soap and water. Although witnessing the formation of a wheal is diagnostic for contact urticaria, a biopsy was performed with the patient’s consent and histopathologic findings were consistent with urticaria (Fig 3).

In the setting of persistent skin peeling and erythema, the patient was treated with topical steroids for a presumed diagnosis of PCD without significant benefit. Given his diagnosis of contact urticaria, the patient was started on a regimen of oral cetirizine titrated up to 40 mg per day that was taken before and after fishing trips with a reported 70% symptomatic improvement. He was counseled on avoidance of triggers as the treatment of choice; however, his enthusiasm for fishing made this the least desirable option. Other options for treatment including omalizumab and dupilumab were considered but declined since they were incompatible with the patient’s occupation as a military pilot.

## Discussion

Contact urticaria is an uncommon form of urticaria that is part of the family of inducible urticaria syndromes and comprises approximately 1% to 8% of occupational skin diseases.^5^ Contact urticaria is most frequently associated with exposures to cow dander, rubber latex, flour and grains, food, and animal products.^5^ Many cases have been reported in bakers, food handlers, health care professionals, and farmers. Although there are also some reported cases of contact urticaria in response to large animal and plant proteins in occupational fishermen, the presence of both urticarial and contact dermatitis symptoms remains uncommon.

In this patient’s case, an accompanying PCD was suspected given his prolonged erythema and scaling. Interestingly, this patient’s reactions were induced only by raw fish, with no reaction noted upon exposure to cooked fish products. This feature implicates sensitivity to a heat-labile fish protein.^5^ Major fish allergens are typically heat stable with parvalbumin representing the most commonly described example protein.^6^ Although there are commercial fish allergens available for prick testing, these extracts can be insufficient for determining IgE reactivity as there are variable amounts of important allergens such as parvalbumin in the samples.^7^ Because of this uncertainty with commercial prick testing options, in-office prick testing with a fresh sample of the patient’s suspected allergen can be accomplished. Prick testing can be done with a prick-by-prick method where the skin is pricked, and allergen rubbed over the area. This can help avoid the risk of anaphylaxis as there is a smaller amount of allergen delivered with this method.^1^ Use of the open application method has less risk of subsequent systemic symptoms and may be the best first step for in-office testing. Open application testing can also be repeated over the span of 24 to 48 hours with re-evaluation in cases where results are initially negative or equivocal.

Unlike other forms of urticaria, contact urticaria syndrome can present with an associated dermatitis with skin peeling and skin changes that extend beyond the 24-hour window associated with wheel formation in urticaria.^2^ Clinical suspicion should remain heightened for contact urticaria syndrome, especially when caring for patients in occupations with repeated exposure to food or animal proteins. Contact urticaria can occur at all stages in a career, including at the onset.^4^ Occupational consequences of contact urticaria, PCD, and contact urticaria syndrome can include increased sickness-related absenteeism, and premature workforce attrition.^8^ It is also likely that the incidence of contact urticaria or PCD are underreported with a possible healthy worker effect as workers may quit before seeking care.^4^

Our patient represents an unusual case of contact urticaria syndrome likely secondary to a heat-labile fish protein. In this case, the recreational nature of the patient’s exposures did not interfere directly with his occupation or continued military service. Although additional treatment options beyond high dose antihistamines and topical corticosteroids were considered, the patient’s military flying status limited his options for treatment with investigative therapies such as omalizumab or dupilumab. These additional treatment options may be beneficial when approaching a case of occupationally related contact urticaria or contact urticaria syndrome, especially if trigger avoidance is not possible.

## Conflicts of interest

None disclosed.
